# Exploratory and confirmatory factor analysis of the questionnaire on Palliative Care for Advanced Dementia (qPAD) using a large sample of staff from Australian residential aged care homes

**DOI:** 10.1111/opn.12505

**Published:** 2022-10-08

**Authors:** Joanne Tropea, Caroline A. Brand, Wen K. Lim, Graham Hepworth, Sue Finch

**Affiliations:** ^1^ Department of Medicine and Aged Care Royal Melbourne Hospital Parkville Victoria Australia; ^2^ Department of Medicine, Royal Melbourne Hospital University of Melbourne Melbourne Victoria Australia; ^3^ Statistical Consulting Centre University of Melbourne Melbourne Victoria Australia

**Keywords:** dementia, factor analysis, nursing attitudes, nursing home care, staff knowledge

## Abstract

**Background:**

The Questionnaire on Palliative Care for Advanced Dementia (qPAD) is increasingly being used to assess residential aged care workers' knowledge and attitudes about palliative care for people with dementia. The qPAD developers performed an exploratory factor analysis and assessed the internal consistency using a small sample.

**Aim:**

The aim of this study was to further assess the structural validity of the qPAD using a large sample of qPAD responses from staff who work in residential aged care homes in Australia.

**Methods:**

Data from 727 care staff who participated in an Australian dementia palliative care training project were used for exploratory factor analyses, assessment of internal consistency, and confirmatory factor analysis of the knowledge test and attitude scale components of the qPAD.

**Results:**

The exploratory factor analysis of the knowledge test produced a four‐factor solution. One item loaded weakly, and four items had cross‐loadings. Factor labels for the knowledge test were difficult to define. Factor analysis of the attitude scale produced a three‐factor structure with good internal consistency—Feeling valued and part of the care team (α = 0.88), Family and team engagement (α = 0.75) and Perceptions and beliefs (α = 0.83). Confirmatory factor analysis indicated improvements in model fit were needed for both the knowledge test and attitude scale.

**Conclusion:**

The findings of this factor analysis differed from the original study. The attitude scale produced a three‐factor structure, but the knowledge test requires further development due to weak and cross‐loadings of several items, inadequate internal consistency of factors and poor model fit.

**Summary statement of implications for practice**

**What does the research add to existing knowledge in gerontology?**

- This study is the first to evaluate construct validity of the Questionnaire on Palliative Care for Advanced Dementia (qPAD) using exploratory and confirmatory factor analysis of a large sample size.
- These findings confirm the attitude scale of the qPAD provides a reliable way to measure nursing home staff attitudes towards caring for people with advanced dementia; however, some of the knowledge test items did not perform well.

**What are the implications of this new knowledge for nursing care with older people?**

- The qPAD attitude scale can be used to assess nursing home nurses' and care workers' perceptions and beliefs, feeling valued and part of a team, and attitudes towards engaging with care team members and with families in advanced dementia care.

**How could the findings be used to influence policy or practice or research or education?**

- Our findings suggest more research is required to further develop and evaluate the knowledge test items of the qPAD before it can be used for education and research purposes.
- The qPAD attitude scale can be used to assess nursing home staff attitudes towards caring for people with advanced dementia, and to measure the impact of targeted interventions and improvement programs.

## 1 INTRODUCTION

In Australia, over half the people in residential aged care homes (or nursing homes) are living with advanced dementia where they will spend the last months or years of life (Australian Institute of Health and Welfare, 2020). Care home staff therefore require knowledge, attitudes and skills that support the palliative and end‐of‐life care needs of people with dementia and their families (van der Steen, 2014). This includes care that focuses on improving quality of life of the person with dementia and their families, through relief from suffering, and ensuring physical, psychosocial and spiritual needs are met (World Health Organisation, 2020). With the growing prevalence of dementia and concomitant demand on aged care services, the need to upskill and train aged care workers in palliative dementia care has been recognised (Worldwide Hospice Palliative Care Alliance [WHPCA], 2015). Such training programs require reliable and valid instruments that measure baseline levels of and change in knowledge, skills and attitudes of care home staff, as well as highlight training gaps and areas of need.

Several instruments have been developed to measure general knowledge and biomedical knowledge of dementia including the different causes of dementia, signs and symptoms, differential diagnoses, and risk factors (Spector, 2013). The Dementia Knowledge Assessment Scale (DKAS) also measures knowledge of psychosocial aspects of communication and care, and has been assessed among lay people, family carers and healthcare workers including people who work in residential aged care (Annear, 2017). Few instruments measure attitudes towards dementia, of which the Dementia Attitude Scale (DAS) is the most widely used (O'Connor, 2010) and has undergone psychometric assessment using samples of university students. However, the DAS validity and reliability have not been assessed among people who work in residential aged care. For our study, we wanted an instrument that had been tested among residential aged care workers and focussed on dementia‐specific palliative care needs. The Questionnaire on Palliative Care for Advanced Dementia (qPAD) fulfilled these criteria (Long, 2012).

The qPAD measures both dementia knowledge and attitudes in the one instrument and was developed in recognition of the need to assess residential aged care staff knowledge and attitudes about dementia end‐of‐life and palliative care. Developed in the United States by clinical and academic nurses with expertise in dementia and a behaviour health practitioner, the qPAD is a 35‐item questionnaire that measures staff knowledge, and attitudes about palliative care for people living with advanced dementia. It is a two‐part instrument with a 23‐item knowledge test and 12‐item attitude scale. The knowledge test items cover caregiving principles related to advanced dementia care such pressure care, pain management and common signs the person is nearing the end of life. Knowledge test scores range from 0 to 23 with higher scores indicating greater knowledge. The attitude scale includes items about perceptions, beliefs and practices when caring for people with advanced dementia. Attitude scale scores range from 12 to 60 with higher scores indicating more positive perceptions and attitudes. A total qPAD score can be derived by adding the knowledge test and attitude scale scores.

The qPAD developers assessed the psychometric properties of the questionnaire in a sample of 85 caregiving staff from four care homes in Arizona, USA (Long, 2012). The authors reported good Cronbach alpha values for the knowledge test (0.81) and attitude scale (0.84). They evaluated the structural validity of the knowledge test and attitude scale items using exploratory factor analysis, a method used to determine the number of distinct constructs (or factors) needed to account for the pattern of correlations among a set of measures (Fabrigar, 2011). In the original study, the exploratory factor analysis produced three factors from the knowledge test items—anticipating needs, preventing negative outcomes and insight and intuition, and three factors from the attitude scale—job satisfaction, perceptions and beliefs, and work setting support of families. Although the findings of this study suggested the two sections provided reliable and valid measures, the authors acknowledged the small sample size as a limitation and stated ‘…the instrument should be used with greater numbers of health caregivers before definitive statements can be made’.

Since its development, the qPAD has been used in several research studies and translated for use in Japan and Taiwan (Agar, 2017; Chen, 2018; Luckett, 2019; Nakanishi, 2016; Nakanishi, 2015). The Japanese study by Nakanishi et al. (2015) reported moderate to good Cronbach's alpha coefficients for the attitude scale items: 0.84 for the 12 items and between 0.55 and 0.79 for the three factors as identified in the original study. Similarly, the Taiwanese study by Chen and colleagues assessed the content validity index and internal consistency and reported good results. Agar et al. (2017) in a cluster randomised controlled trial of facilitated family case conferencing, used the qPAD to assess aged care workers' baseline and change in dementia palliative care knowledge and attitudes. They analysed baseline qPAD data to explore associations between qPAD scores and aged care facility/personal characteristics, and found being a nursing manager, registered nurse or enrolled nurse, and having a preferred language of English were associated with more favourable knowledge test scores; and having tertiary level education and greater experience in dementia care was associated with favourable attitudes (Luckett, 2019). None of these studies evaluated the structural validity of the qPAD using confirmatory factor analysis.

## 2 AIM

The aim of this study was to assess the psychometric properties, including structural validity and internal consistency of the qPAD using a large sample of Australian residential aged care staff responses, and to compare these findings to the factor structure defined by the USA developers.

## 3 METHODS

### 3.1 Study design and subjects

The present study was part of a larger study—the IMproving Palliative care Education and Training Using Simulation in Dementia (IMPETUS‐D) study: a cluster randomised controlled trial (Tropea, 2019). The IMPETUS‐D project was supported by the Australian Government Dementia and Aged Care Grant opportunity 1, with ethical approval received by the Melbourne Health Human Research Ethics Committee (HREC/17/MH/336).

The qPAD was used in the above‐mentioned study as a secondary outcome measure of dementia knowledge and attitudes. It was chosen for the following two reasons: (i) it was developed specifically for direct care staff who work in residential aged care (also known as nursing homes and long‐term care), and (ii) it measures staff knowledge of and attitudes about palliative and end‐of‐life care for people with dementia which was the focus of the IMPETUS‐D project.

The study involved 24 residential aged care homes of a single private provider in Sydney, Melbourne and Adelaide, Australia. To be eligible for inclusion in the study, care homes had to have a minimum of 20 residents with a diagnosis of dementia and high care needs living permanently in the homes.

After recruitment of residential aged care homes, the qPAD data were collected to determine care home staff baseline level of knowledge and attitudes towards palliative dementia care. All direct care staff, defined as nurses and personal carers/attendants in nursing, who were working at the care homes, were invited to participate in the survey. Participation was voluntary, and staff were required to give informed consent prior to completion of the survey.

### 3.2 Measurements

The survey instrument collected participant characteristics, including position, age, gender, education level, years worked in aged care, previous training in dementia and previous training palliative care. This was followed by qPAD instrument measures. For each item of the knowledge test, respondents were asked to select from Agree, Disagree or Don't know. A score of 1 is allocated to each correct response and 0 for an incorrect or ‘Don't know’ response. Knowledge test scores range from 0 to 23 with higher scores indicating greater knowledge. For the attitude scale, respondents were asked to rate each item using a five‐point scale of 1 Strongly disagree, 2 Disagree, 3 Neutral, 4 Agree and 5 Strongly agree. Attitude scale scores range from 12 to 60 with higher scores indicating more positive perceptions and attitudes. A total qPAD score is derived by adding the knowledge test and attitude scale scores. However, we did not use the total score for the purposes of the factor analysis.

### 3.3 Data collection

Surveys were sent to all direct care staff (*N* = 1947) via email with a unique survey link generated by Research Electronic Data Capture (REDCap) (Harris, 2009). To increase participation at facilities with low response rates, personalised letters of invitation with hard copy surveys and reply‐paid envelopes enclosed were also sent.

Responses were collected directly via REDCap, or hard copy responses were entered into REDCap by members of the research team. Data were then exported for analysis, and responses were randomly assigned into two datasets of approximately equal size for exploratory factor analysis sample (*N* = 364) and confirmatory factor analysis sample (*N* = 363). Only complete responses were included in the analysis. Statistical analysis was conducted using Stata 15.1 MP (StataCorp., 2017).

### 3.4 Data analysis

#### 3.4.1 Exploratory factor analysis (EFA)

Descriptive statistics were calculated, including mean and standard deviation of knowledge test, attitude scale and total qPAD scores, the proportion correct for each knowledge test item and the mean for each attitude scale item. EFA were conducted to assess the structure of the knowledge test and attitudes scale. Initial exploration of the Australian data included replicating the principal factor analysis methods used in the USA study, retaining three factors, and employing an oblique rotation model for the knowledge test and orthogonal rotation model for the attitude scale. The findings are available as Tables S1 and S2.

However, the original qPAD factor analysis used traditional methods based on Pearson correlations and the assumption that the variables are continuous and follow a normal distribution. The EFA presented in this study differs from the original study. We have acknowledged the qPAD items are not continuous variables—the knowledge test uses a binary measure, and the attitude scale uses a five‐point Likert scale which we consider ordinal. We used a more optimal approach to the factor analysis methods for non‐continuous variables by using tetrachoric correlations for the knowledge test and polychoric correlations for the attitude scale to estimate the correlation had the measurement scales been continuous (Holgado–Tello, 2008). We used Promax oblique rotation to allow for correlated latent factors.

Prior to extraction of factors, the suitability of the data for factor analysis was assessed and met using Bartlett's chi‐square test of sphericity (*p* < 0.05) and Kaiser–Meirer–Olgin (KMO > 0.5) measure of sampling adequacy. Scree plots were examined looking for an ‘elbow’ or distinct break in the slope of the scree plot (Cattell, 1966), and parallel analysis (Horn, 1965) was used to determine the number of factors to retain in the models. The exploratory analysis employed principal factor analysis with Promax oblique rotation. The internal consistency of the knowledge test, attitude scale and each of the scales' factors was assessed using Cronbach's alpha coefficient with an alpha equal to or greater than 0.70 considered satisfactory (Nunnally, 1978). Additional exploration was conducted retaining up to six factors for the knowledge test and up to five factors for the attitude scale.

The structure models were evaluated for the following: (i) number of items with salient loadings defined as loadings ≥0.40, (ii) number of items that have cross‐loadings defined as loadings ≥0.40 onto more than one factor, (iii) internal consistency ≥0.70 and (iv) minimum of two items load onto a factor to allow meaningful interpretation for factors to be descriptively labelled.

#### 3.4.2 Confirmatory factor analysis (CFA)

Confirmatory factor analysis was performed using structural equation modelling with a maximum likelihood parameter estimation method on the confirmatory factor sample using the tetrachoric (knowledge test) and polychoric (attitude scale) correlations of the items identified from the best model in the EFA. Standardised factor loadings and corresponding p values were examined (*p* < 0.05). The equation‐level fit was assessed by examining *R*‐squared values for each item, and overall model‐level fit was evaluated using the following goodness‐of‐fit measures indicating acceptable fit: (i) chi‐square test with a nonsignificant chi‐square (*p* > 0.95); (ii) the comparative fit index (CFI) ≥0.90; (iii) the standardised root mean square residual (SRMR) ≤0.08; (iv) the root mean square error of approximation (RMSEA) values <0.05 suggesting good fit and values between 0.05 and 0.08 suggesting reasonable fit; and (v) the Tucker‐Lewis Index (TLI) (≥0.95) (Jackson, 2009). CFA using the factor structures from the original EFA was then performed, and goodness‐of‐fit statistics from both models were compared.

## 4 RESULTS

### 4.1 Study participants

A total of 727 (37%) direct care staff from 24 care homes participated in the questionnaire. Table 1 summarises the participants' characteristics of the total sample, including the average qPAD scores. Most participants were personal carers (70%), female (85%), and 71% had worked less than 10 years in aged care homes. The mean (standard deviation) knowledge test, attitude scale and total qPAD scores were 14.9 (3.3), 44.7 (7.7) and 59.5 (8.7), respectively.

**TABLE 1:** Characteristics of total sample (*N* = 727)

Characteristics, *n* (%)	
Position	
Personal carer/Attendant in nursing	516 (71)
Registered nurse	172 (24)
Enrolled nurse	26 (4)
Other^a^	13 (1.5)
Age in years, mean (SD)	40.3 (12.6)
Female	618 (85)
Highest level of education	
Secondary school	182 (25)
Post‐secondary, non‐tertiary	158 (22)
University degree	326 (45)
Other	16 (2)
Missing	45 (6)
Years worked in residential aged care	
Less than 5 years	323 (44)
5–10 years	196 (27)
11–15 years	92 (13)
More than 15 years	110 (15)
Missing	6 (1)
Previous training in dementia	312 (43)
Previous training in palliative care	265 (36)
qPAD score, mean (SD)	
Knowledge test	14.9 (3.3)
Attitude scale	44.7 (7.7)
Total score	59.5 (8.7)

Abbreviations: SD, standard deviation.

^a^ 2 Lifestyle/activity coordinators, 11 missing.

### 4.2 Exploratory factor analysis—knowledge test

Examination of the scree plot of eigenvalues and parallel analysis of the knowledge test items suggested a three‐factor and four‐factor model, respectively (Supplementary Information S3). The three‐ and four‐factor models were assessed against the stated criteria.^1^ All items except item 11 (highest factor loading 0.391) loaded satisfactorily onto the four‐factor structure. Item 12 (highest factor loading 0.285) and item 7 (highest factor loading 0.395) did not load well onto any factors of the three‐factor model. Both models had an item cross‐load onto more than one factor. The four‐factor Promax rotated model was selected as the best EFA model and has been presented in Table 2. The four factors accounted for 55% of the total variance, and the internal consistency of the four‐factor model was 0.75, 0.49, 0.59 and 0.40. Factor 1 was the only factor to meet the internal consistency criteria (≥0.70).

**TABLE 2:** Exploratory factor analysis with Promax oblique loadings of qPAD knowledge test (*N* = 364)

Item #	Item description	Factor 1	Factor 2	Factor 3	Factor 4
1	The best way to prevent weight loss for persons with advanced dementia is to keep them on their special medical diets (e.g. low fat, cardiac and renal).	**0.4321**	0.0760	−0.2303	−0.1729
4	Since persons with advanced dementia are so impaired, it is not likely that they are depressed.	**0.6942**	−0.1128	−0.0793	0.0457
5	One benefit of advanced dementia is that people no longer have pain.	**0.8211**	−0.1744	−0.0346	0.0484
9	Persons with advanced dementia can reposition themselves easily in their chairs.	**0.6836**	−0.0978	**0.4090**	−0.0868
11	The sounds of music, meal service and conversations during dining do not generally pose problems for people with advanced dementia.	0.3916	0.2889	0.1727	−0.3642
13	Physical restraints decrease the chance that a person with advanced dementia will fall.	**0.5696**	0.1875	−0.0611	0.0552
14	When people ‘call out’ over and over again, it is best to not worry about this behaviour because this is a common occurrence for persons with advanced dementia.	**0.7294**	0.1711	−0.1061	0.0679
15	Persons with advanced dementia will never experience boredom.	**0.8949**	−0.0027	−0.0562	0.1753
19	If a person with advanced dementia is unable to sleep at night, a sleeping medication should be considered first.	**0.4987**	0.3533	−0.1197	−0.0745
20	When persons with advanced dementia spit out their food, it is because they are not hungry.	**0.8352**	−0.0448	0.0035	0.2901
23	When persons with advanced dementia rapidly become more confused or display changes in behaviour, it is likely that their dementia is getting worse.	**0.4978**	0.1137	**−0.4525**	−0.0418
2	It is possible to prevent pressure ulcers in persons with advanced dementia.	0.0703	**0.7286**	−0.0526	−0.0199
3	It is possible to prevent weight loss in most persons with advanced dementia.	−0.3778	**0.5250**	−0.2672	0.0801
6	When a person is resistive to ‘hands‐on’ care, it is best to stop what you are doing and come back later to try to complete the task.	−0.0116	**0.6682**	**0.4751**	−0.1342
10	Although persons with advanced dementia are incontinent, it is still possible to toilet them.	−0.0017	**0.7493**	−0.0363	0.1315
16	If persons with advanced dementia resist (e.g. hit, bite and kick) a brief change, it may be due to invasion of privacy.	0.0077	**0.7334**	0.1883	0.0323
8	Persons with advanced dementia cannot verbally tell us when they are hungry or thirsty.	0.0187	0.0876	**0.8676**	0.0355
21	Persons with advanced dementia really cannot convey or relate to caregivers if they are hungry, have pain or need to use the bathroom.	0.3125	0.0278	**−0.6553**	0.0008
22	Persons with advanced dementia can fatigue or tire easily, and as a result, they usually need to lie down frequently.	−0.0157	0.2099	**0.6280**	0.2799
7	Persons with advanced dementia should take showers just as other persons do.	0.1874	−0.1146	0.1899	**−0.6684**
12	Persons with advanced dementia typically die from some sort of infection, such as pneumonia or a urinary tract infection.	−0.1549	0.1834	0.0672	**0.4282**
17	Persons with advanced dementia should get pain medications around‐the‐clock, when needed.	**0.4075**	0.1250	0.1643	**0.7811**
18	‘Anticipation of need’ refers to addressing the needs of persons with advanced dementia through a daily schedule established by the facility where they live.	−0.2917	0.1956	−0.2041	**−0.7267**

*Note*: Method of extraction: principal factors. Salient loadings ≥0.40 in boldface.

We were unable to label the four factors as items that loaded onto each factor covered a mix of themes and varied to the USA findings, for example the ‘Factor 1‐ Anticipating needs’ items loaded onto factors 1 and 4 of this study, ‘Factor 2 ‐ Preventing negative outcomes’ items loaded across factors 1 and 4, and items from ‘Factor 3 ‐ Insight and intuition’ loaded across factors 2, 3 and 4 of our study.

Items 6, 10, 2 and 5 had high mean values of 0.91, 0.87, 0.87 and 0.86, respectively, indicating almost all participants answered these items correctly, while items 18 and 1 had low mean values of 0.11 and 0.30, respectively, indicating most participants had a low understanding of these items.

### 4.3 Confirmatory factor analysis—knowledge test

The CFA of knowledge test items assessed the fit of the above four‐factor structure using the CFA sample (*N* = 363). Standardised factor loadings for twenty (83%) items reached statistical significance (*p* < 0.05), while factor loadings of items 3, 7 and 22 were weak and did not reach statistical significance (Table 3). Equation‐level goodness of fit showed items 3 and 22 had the lowest *R*‐squared values, each with values less than 1% and items 2, 7, 18 had values less than 5%, while item 21 had the highest *R*‐squared value of 89% (See Supplementary Information S3). The chi‐square test (Chi^2^ = 419.90, *p* < 0.001), CFI (CFI = 0.80) and TLI (TLI = 0.78) indicated a poor level of fit, while the RMSEA (RMSEA = 0.05, *p* = 0.573) and SRMR (SRMR = 0.06) indicated acceptable fit (Table 4).

**TABLE 3:** Loading estimates from the confirmatory factor analysis of qPAD knowledge test (*N* = 363) four‐factor model using structural equation model

Item no.	Knowledge test items	Standardised coefficient
Factor 1		
1	The best way to prevent weight loss for persons with advanced dementia is to keep them on their special medical diets (e.g. low fat, cardiac and renal).	0.26
4	Since persons with advanced dementia are so impaired, it is not likely that they are depressed.	0.40
5	One benefit of advanced dementia is that people no longer have pain.	0.63
9	Persons with advanced dementia can reposition themselves easily in their chairs.	0.43
11	The sounds of music, meal service and conversations during dining do not generally pose problems for people with advanced dementia.	0.35
13	Physical restraints decrease the chance that a person with advanced dementia will fall.	0.52
14	When people ‘call out’ over and over again, it is best to not worry about this behaviour because this is a common occurrence for persons with advanced dementia.	0.67
15	Persons with advanced dementia will never experience boredom.	0.67
19	If a person with advanced dementia is unable to sleep at night, a sleeping medication should be considered first.	0.59
20	When persons with advanced dementia spit out their food, it is because they are not hungry.	0.56
23	When persons with advanced dementia rapidly become more confused or display changes in behaviour, it is likely that their dementia is getting worse.	0.30
Factor 2		
2	It is possible to prevent pressure ulcers in persons with advanced dementia.	0.19
3	It is possible to prevent weight loss in most persons with advanced dementia.	0.08*
6	When a person is resistive to ‘hands‐on’ care, it is best to stop what you are doing and come back later to try to complete the task.	0.24
10	Although persons with advanced dementia are incontinent, it is still possible to toilet them.	0.36
16	If persons with advanced dementia resist (e.g. hit, bite and kick) a brief change, it may be due to invasion of privacy.	0.40
Factor 3		
8	Persons with advanced dementia cannot verbally tell us when they are hungry or thirsty.	0.42
21	Persons with advanced dementia really cannot convey or relate to caregivers if they are hungry, have pain or need to use the bathroom.	0.95
22	Persons with advanced dementia can fatigue or tire easily, and as a result, they usually need to lie down frequently.	0.10*
Factor 4		
7	Persons with advanced dementia should take showers just as other persons do.	0.11*
12	Persons with advanced dementia typically die from some sort of infection, such as pneumonia or a urinary tract infection.	0.25
17	Persons with advanced dementia should get pain medications around‐the‐clock, when needed.	0.31
18	‘Anticipation of need’ refers to addressing the needs of persons with advanced dementia through a daily schedule established by the facility where they live.	0.22

* *p* > 0.05.

**TABLE 4:** Goodness‐of‐fit statistics for the confirmatory factor analysis of the knowledge test (*N* = 363)

Fit statistic	Four‐factor model from current study	Three‐factor model from original study
chi‐square (df) *p* value	419.900 (224) 0.000	Not reported because the fitted model is not full rank
CFI	0.804	1.000
TLI	0.778	‐
RMSEA (90% CI)	0.049 (0.042–0.056)	‐
SRMR	0.062	0.068

### 4.4 Exploratory factor analysis—Attitude scale

The screen test and parallel analysis supported retaining three factors for the EFA of the attitude scale items (See Supplementary Information S3). Table 5 outlines the results of the attitude scale three‐factor solution with Promax oblique rotation, each factor accounting for 71%, 19% and 10% of the variance. Item 9 of the attitude scale had cross‐loadings with similar loadings across factors one (0.46) and two (0.42). Overall, this solution demonstrated good structure with all item loadings being greater than 0.4, and good internal consistency with alpha coefficients of 0.88, 0.75 and 0.83 for factors 1, 2 and 3, respectively.

**TABLE 5:** Exploratory factor analysis with Promax oblique rotation loadings of qPAD Attitude Scale (*N* = 364)

Item #	Item description	Factor 1	Factor 2	Factor 3
7	I am regularly included in the care‐planning for persons with advanced dementia.	**0.7268**	−0.0092	0.2117
8	My supervisor and team regularly listen to me regarding suggestions for persons with advanced dementia.	**0.9529**	−0.1262	0.0955
9	On most days, I am satisfied with my job of caring for persons with advanced dementia.	**0.4606**	**0.4160**	−0.1778
10	My input and opinion are valued regarding the needs of persons with advanced dementia.	**0.9585**	−0.0616	0.0316
11	On most days, I feel I'm part of the care team.	**0.6657**	0.2700	−0.0788
4	Families are given consistent information about the consequences of their end‐of‐life care decisions.	−0.1663	**0.7285**	0.2828
5	Families are regularly included in ongoing discussions regarding advanced dementia care needs for their loved ones.	−0.0796	**0.8430**	0.0245
6	I frequently talk with my teammates about how we can change and improve the care for persons with advanced dementia.	0.2481	**0.4365**	0.0436
12	I enjoy providing care for persons who have advanced dementia.	0.2271	**0.5962**	−0.2016
1	I believe my work experience enables me to discuss advanced dementia care with families.	0.0275	0.0208	**0.9310**
2	I believe my education enables me to discuss advanced dementia care with families.	0.0515	−0.0224	**0.9484**
3	I believe it is important that caregivers provide families with information about end‐of‐life decisions.	0.0975	0.1965	**0.4718**

*Note*: Method of extraction: principal factors. Salient loadings ≥0.40 in boldface.

Five items loaded onto factor 1 defined as ‘Feeling valued and part of the care team’, four items loaded onto factor 2 defined as ‘Family engagement’, and the remaining three items loaded onto factor 3 which we defined as ‘Perceptions and beliefs’. In our study, items 10 and 12 loaded onto factor 2—Family engagement, whereas in the USA study these items loaded onto factor 1 which was labelled ‘Job satisfaction’. Finally, mean values of items ranged from 3.2 (item 1 and item 2) to 4.2 (item 12).

### 4.5 Confirmatory factor analysis—Attitude scale

The CFA of attitude scale items assessed the fit of the three‐factor structure using the CFA sample (*N* = 363). All standardised factor loadings were statistically significant (*p* < 0.05) (Table 6). Equation‐level goodness of fit showed item 3 had the lowest *R*‐squared value of 25% and items 1 and 2 had the highest *R*‐squared values of 84% and 85%, respectively (See Supplementary Information S3). The model chi‐squared test (Chi^2^ = 364.67, p < 0.001), CFI (CFI = 0.86), TLI (TLI = 0.82) and RMSEA (RMSEA = 0.13) values indicated the model had less than acceptable fit, while the SRMR (SRMR = 0.08) indicated acceptable fit (Table 7).

**TABLE 6:** Loading estimates from the confirmatory factor analysis of qPAD Attitude Scale (*N* = 363) using structural equation modelling

Item no.	Attitude scale statements	Standardised coefficient
Factor 1—Feeling valued and part of the care team		
7	I am regularly included in the care planning for persons with advanced dementia.	0.64
8	My supervisor and team regularly listen to me regarding suggestions for persons with advanced dementia.	0.81
9	On most days, I am satisfied with my job of caring for persons with advanced dementia.	0.62
10	My input and opinion are valued regarding the needs of persons with advanced dementia.	0.86
11	On most days, I feel I'm part of the care team.	0.79
Factor 2—Family engagement		
4	Families are given consistent information about the consequences of their end‐of‐life care decisions.	0.65
5	Families are regularly included in ongoing discussions regarding advanced dementia care needs for their loved ones.	0.73
6	I frequently talk with my teammates about how we can change and improve the care for persons with advanced dementia.	0.65
12	I enjoy providing care for persons who have advanced dementia.	0.56
Factor 3—Perceptions and beliefs		
1	I believe my work experience enables me to discuss advanced dementia care with families.	0.92
2	I believe my education enables me to discuss advanced dementia care with families.	0.92
3	I believe it is important that caregivers provide families with information about end‐of‐life decisions.	0.50

**TABLE 7:** Goodness‐of‐fit statistics for the confirmatory factor analysis of the attitude scale (*N* = 363)

Fit statistic	Four‐factor model from current study	Three‐factor model from original study
chi‐square (df) *p* value	364.667 (51) 0.000	346.615 (51) 0.000
CFI	0.859	0.867
TLI	0.817	0.828
RMSEA (90% CI)	0.130 (0.118–0.143)	0.126 (0.114–0.139)
SRMR	0.081	0.075

## 5 DISCUSSION

This study builds on the psychometric assessment of the qPAD instrument reported previously by Long et al. (2012). Using a large sample of qPAD responses from over 700 Australian residential aged care workers, our exploratory and confirmatory factor analyses showed some differences and similarities to the original USA study. These will now be discussed.

In our study, EFA of the qPAD knowledge test, a four‐factor solution was considered the best model compared to the three‐factor model reported in the USA study (See Supplementary Tables S1 and S2). Our model had salient factor loadings, defined as loadings ≥0.40, for all items except item 11, whereas in the USA study six items (items 11, 21, 3, 6, 15 and 17) did not meet this criterion. The internal consistency was good for two factors and poor for one factor in the previous study, whereas it was good for one and poor for three factors in the current study. In addition, unlike the USA study we were unable to label the constructs of the knowledge test as no clear themes were definable.

In both studies, the EFA of the attitude scale yielded a three‐factor solution, and the same three items of the attitude scale loaded onto the factor labelled ‘Perceptions and beliefs’. However, items 6 and 12 loaded onto different factors across the two studies The USA study reported good internal consistency for only one of the attitude scale factors (factor 1 alpha level of 0.90), whereas our study demonstrated good internal consistency across all three factors suggesting our factor structure was more suitable.

The observed differences in factor structure may be due to several reasons, including differences in the characteristics of the samples, and cultural differences between the United States and Australia. It may also be that there have been changes over the past 10 years in knowledge and attitudes about dementia in the cohorts of interest, and more generally. Our study of 727 participants from 24 care homes across three Australian cities is potentially more representative of this cohort compared to the USA study with a sample of 85 staff from three care homes in one American state. The small sample size in the USA study is one of the main limitations. The sample size of our study met the factor analysis criteria described by Fabrigar and Wegener (2011), whereas the USA study did not, specifically the recommendation of a sample size of at least 200 under moderately good conditions (defined as communalities of 0.40–0.70 and at least three items loading on each factor) and a sample size of at least 400 under poor conditions (defined as communalities lower than 0.40 and some factors with only two measured variables) (Fabrigar, 2011). Given our study met these criteria, our factor loading matrix for the knowledge test and attitude scale are likely to represent a more stable solution. A further difference among the two studies was the use of different analytical methods. The USA study used Pearson correlations, whereas our study used tetrachoric and polychoric correlations for binary and ordinal data, respectively. The methods we used are considered the more suitable method for factor analysis of non‐continuous variables as is the case for the qPAD variables (Holgado–Tello, 2008; Norris, 2010).

In the present study, the confirmatory factor analyses of the four‐factor structure of the knowledge test and the three‐factor structure of the attitude scale were conducted. The results showed inadequate structure for the knowledge test according to the range of criteria we considered. The results for the attitude test were better, although not all of the goodness‐of‐fit criteria were satisfied. The modification indices from the CFA (available on request) suggested that inclusion of correlated errors could improve the model fit.

The results should be interpreted within the limitations of the study. This study used a purposive sampling approach and achieved a 37% response rate. The sample consisted of personal carers and nurses who worked in 24 Australian residential aged care facilities of one aged care provider, which may limit the generalisability of our findings. We included only nurses and personal carers as they provide the bulk of the direct care and were the target group for the larger study. We did not include ancillary staff, allied health or visiting general practitioners. Further those who responded may not be representative of the larger cohort, although the characteristics of the sample indicated representation of both direct care worker groups and diversity in terms of work experience and education levels.

The current study was limited to evaluating the psychometric properties of the qPAD using factor analysis and by assessing internal consistency. Further development of a knowledge measure may require some fundamental item and scale development work. Evaluation of convergent and/or discriminant validity of the attitude scale would further our understanding.

Since the qPAD's development over 10 years ago, there has been a growing body of literature about the palliative care needs of people living with dementia and their families. These include the European Association of Palliative Care's White Paper and the joint report by Palliative Care Australia and Dementia Australia (Palliative Care Australia, 2018; van der Steen, 2014). As highlighted in the reports, although the principles of palliative care are similar for all terminal conditions, people with dementia and their families have specific care needs that address issues unique to dementia including the unpredictable disease trajectory, the severe cognitive impairment that affects the person's decision‐making capacity and verbal communication, the behavioural and psychological symptoms of dementia (BPSD), and the impact dementia can have on caregiver burden. One of the major limitations of the qPAD is the lack of a conceptual or theoretical framework. Future work should consider frameworks that could support survey development. This along with the growing body of literature and expert‐informed best practice recommendations could potentially be used to strengthen the qpad through revision, development and further assessment of items. For example, the knowledge test could include items about setting care goals and advance care planning, the typical trajectory of dementia and challenges of prognostication, the risks associated with hospitalisation, best practice to reduce burdensome and futile interventions, and optimal management of common symptoms at the end of life such as pain, dyspnoea and agitation, and why a different approach is often needed for people with dementia.

With the ageing population and growing prevalence in dementia, a tool that comprehensively assesses nurses' and other aged care staff knowledge of and attitudes towards dementia‐specific palliative care would be valuable, and could be used to understand the areas of knowledge and attitudes that need improvement, as well as evaluate the impact of targeted interventions. However, our findings indicated further development and evaluation of the qPAD is required before it can be used for education and research purposes.

## 6 CONCLUSIONS AND IMPLICATIONS

In this study, exploratory factor analysis of the qPAD resulted in a four‐factor structure of the knowledge test and a three‐factor structure of the attitude scale. The factor structure and some of the factor labels differed to those proposed in the original study. This, together with confirmatory factor analyses suggested a need for redevelopment of the knowledge test items and further validation for the attitude scale. We therefore recommend revision of the qPAD, with a particular focus on knowledge test items.

## FUNDING INFORMATION

This work was supported by funding from the Australian Government Department of Health under the Dementia and Aged Care Services Grant Opportunity 1. Joanne Tropea received support from Melbourne Ageing Research Collaboration (MARC) PhD Scholarship. The funders do not have any input in the study design, analysis or manuscript development.

## CONFLICT OF INTEREST

The authors declare no conflicts of interest.

## Supporting information


Appendix S1

Table S1

Table S2

## Data Availability

Data will be available after analyses is finalised and report / publication has been submitted and approved. Unidentifiable individual participant data and related data dictionaries will be available. Access is subject to approval by the Principal Investigator Professor Wen Kwang Lim.

## Endnote

^1^
Additional factor analyses retaining one, two, five and six factors for the knowledge test and one, two, four and five factors for the attitude scale did not achieve better results.
