# Supplementary material for: Exploratory and confirmatory factor analysis of the questionnaire on Palliative Care for Advanced Dementia (qPAD) using a large sample of staff from Australian residential aged care homes
Source: Int J Older People Nurs. 2022 Oct 8;18(1):e12505. doi: 10.1111/opn.12505 (PMC10078386; doi:10.1111/opn.12505)
Supplement: Supplementary file 1 — Appendix S1 [file OPN-18-0-s002.docx]

**S3: Supplementary information for EFA and CFA of qPAD**

# Supplementary data – Knowledge test EFA

1. Bartlett’s test and KMO

Determinant of the correlation matrix

Det = 0.024

Bartlett test of sphericity

Chi-square = 1323.354

Degrees of freedom = 253

p-value = 0.000

H0: variables are not intercorrelated

Kaiser-Meyer-Olkin Measure of Sampling Adequacy

KMO = 0.751

1. Scree plot


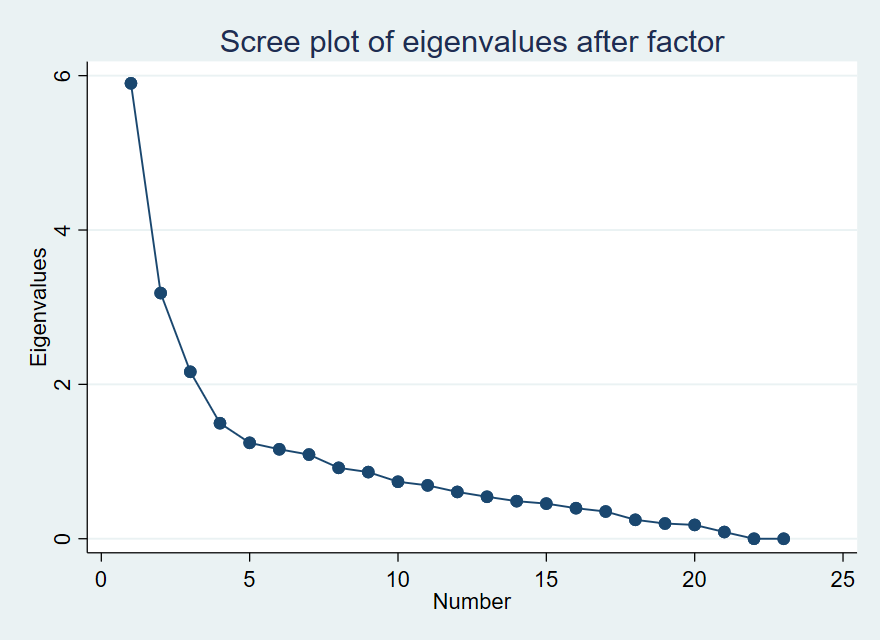


1. Parallel analysis

100 iterations, using the mean estimate

----------------------------------------------------------------

Component Adjusted Unadjusted Estimated

or Factor Eigenvalue Eigenvalue Bias

-----------------------------------------------------------------

1 **3.2010729** 3.7203105 .51923752

2 **1.8771377** 2.2426289 .36549115

3 **1.3610949** 1.6778051 .31671023

4 **1.0546126** 1.2956638 .2410512

5 .93544542 1.1617943 .22634888

6 .90549947 1.1018588 .19635928

7 .86804314 1.0509117 .1828686

8 .866449 .99323106 .12678206

9 .82524655 .93815074 .11290419

10 .76497174 .85054768 .08557594

11 .7912944 .83203536 .04074097

12 .80879025 .77979438 -.02899587

13 .78007781 .73095964 -.04911816

14 .79451103 .71041692 -.08409411

15 .80799801 .7034253 -.10457271

16 .82129749 .66595622 -.15534127

17 .83978383 .62968717 -.21009666

18 .79922655 .56929251 -.22993404

19 .80104974 .55111 -.24993974

20 .7944683 .51514132 -.27932698

21 .75844797 .45726987 -.3011781

22 .76731567 .43367676 -.33363891

23 .77616539 .38833197 -.38783342

------------------------------------------------------------------

Criterion: retain adjusted components > 1

1. **Exploratory factor analysis of the knowledge test retaining three factors using Promax oblique rotations**

Factor analysis/correlation Number of obs = 364

Method: principal factors Retained factors = 3

Rotated factor loadings (pattern matrix) and unique variances

------------------------------------------------------------------------

Variable | Factor1 Factor2 Factor3 | Uniqueness

---------------+------------------------------------+------------------

q1 | **0.4667** -0.3097 0.0619 | 0.6159

q2 | 0.1395 -0.0949 **0.7021** | 0.4393

q3 | -0.3809 -0.2311 **0.5744** | 0.6025

q4 | **0.6625** -0.0416 -0.0859 | 0.5636

q5 | **0.7877** 0.0047 -0.1526 | 0.4055

q6 | 0.1418 0.3355 **0.5216** | 0.5718

q7 | **0.3956** -0.1790 -0.3304| 0.7224

q8 | 0.1087 **0.8197** -0.0560 | 0.3537

q9 | **0.7445** 0.3390 -0.1927| 0.4539

q10 | 0.0261 0.0009 **0.7618** | 0.4110

q11 | **0.5459** -0.0498 0.1434 | 0.6377

q12^ | -0.2578 0.2844 0.2848 | 0.7663

q13 | **0.5652** -0.0346 0.2025 | 0.5858

q14 **| 0.7151** -0.0682 0.1971 | 0.3711

q15 | **0.8378** 0.0451 0.0508 | 0.2911

q16 | 0.0873 0.1564 **0.6801** | 0.4795

q17 | 0.2053 **0.5695** 0.3071 | 0.5472

q18 | -0.0794 **-0.5924** 0.0219 | 0.6623

q19 |  **0.5421** -0.1672 0.3359 | 0.4616

q20 | **0.7463** 0.1643 0.0325 | 0.4528

q21 | 0.2457 -**0.6080** 0.1383 | 0.4851

q22 | -0.0123 **0.7235** 0.1711 | 0.4355

q23 |  **0.4735** **-0.4466** 0.1726 | 0.4371

------------------------------------------------------------------------

Salient loadings (≥0.4) are in boldface. ^item 12 did not have a salient loading.

Factor rotation matrix

---------------------------------------------------

| Factor1 Factor2 Factor3

---------------+-----------------------------------

Factor1 | 0.9629 -0.3405 0.3949

Factor2 | 0.0052 0.8106 0.6126

Factor3 | -0.2697 -0.4764 0.6847

---------------------------------------------------

**5. Exploratory factor analysis of the qPAD Knowledge Test comparing the Australian study four-factor structure (N=343) and USA study three-factor structure (N=85)**

|  | **Australian study Promax oblique loadings; principal factors** | | | | **USA study factor loadings** | | |
| --- | --- | --- | --- | --- | --- | --- | --- |
| **Item number*** | **Factor 1**  **α=0.75** | **Factor 2**  **α=0.49** | **Factor 3**  **α=0.59** | **Factor 4**  **α=0.40** | **Factor 1**  **α=0.75** | **Factor 2**  **α=0.73** | **Factor 3**  **α=0.58** |
| 1 | 0.43 |  |  |  | 0.67 |  |  |
| 2 |  | 0.73 |  |  |  | 0.44 |  |
| 3 |  | 0.53 |  |  |  | 0.25^ |  |
| 4 | 0.69 |  |  |  |  | 0.50 |  |
| 5 | 0.82 |  |  |  |  | 0.72 |  |
| 6 |  | 0.67 |  |  |  | 0.27^ |  |
| 7 |  |  |  | 0.66 | 0.55 |  |  |
| 8 |  |  | 0.87 |  |  |  | 0.48 |
| 9 | 0.68 |  |  |  | 0.40 |  |  |
| 10 |  | 0.75 |  |  |  | 0.52 |  |
| 11 | 0.39 |  |  |  | 0.34^ |  |  |
| 12 |  |  |  | 0.43 |  |  | 0.62 |
| 13 | 0.57 |  |  |  |  | 0.51 |  |
| 14 | 0.73 |  |  |  | 0.52 |  |  |
| 15 | 0.89 |  |  |  |  | 0.38^ |  |
| 16 |  | 0.73 |  |  |  |  | 0.43 |
| 17 |  |  |  | 0.78 |  | 0.29^ |  |
| 18 |  |  |  | 0.73 | 0.57 |  |  |
| 19 | 0.50 |  |  |  |  | 0.52 |  |
| 20 | 0.84 |  |  |  |  | 0.42 |  |
| 21 |  |  | 0.66 |  | 0.25^ |  |  |
| 22 |  |  | 0.63 |  |  |  | 0.55 |
| 23 | 0.50 |  |  |  | 0.77 |  |  |

*Note item number 1 corresponds to item number 3 of USA study, item 2 corresponds to item 4, item 3 to item 5 etc.

^Loadings <0.40 indicating did not meet the criteria set for salient loadings.

# Supplementary data – Knowledge test CFA

Equation-level goodness of fit

---------------------------------------------------------------------------------------------------------

| Variance |

depvars | fitted predicted residual | R-squared mc mc2

----------------+-------------------------------------------+-------------------------------------------

observed | |

q15 | .1167649 .0526094 .0641555 | .4505583 .6712364 .4505583

q20 | .1304707 .0406648 .089806 | .3116775 .5582808 .3116775

q5 | .0958951 .037904 .057991 | .3952657 .6287016 .3952657

q14 | .1147463 .0520873 .0626589 | .453935 .673747 .453935

q4 | .2122806 .0343552 .1779254 | .1618384 .4022915 .1618384

q9 | .1718158 .0318559 .13996 | .1854071 .4305892 .1854071

q13 | .2020354 .0539441 .1480913 | .2670032 .5167235 .2670032

q19 | .2222222 .0762179 .1460043 | .3429807 .5856456 .3429807

q1 | .2143903 .0148771 . 1995132 | .0693928 .2634251 .0693928

q11 | .2393279 .0300679 .20926 | .1256346 .3544497 .1256346

q23 | .213343 .0195314 .1938116 | .0915494 .3025713 .0915494

q10 | .0937246 .0120108 .0817139 | .1281494 .3579796 .1281494

q16 | .2164394 .0346389 .1818004 | .1600399 .4000499 .1600399

q2 | .0893382 .003367 .0859712 | **.0376877 .**1941332 .0376877

q6 | .0826446 .0045864 .0780582 | .0554952 .2355742 .0554952

q3 | .2193991 .0014502 .2179489 | **.0066099** .0813012 .0066099

q8 | .2203553 .0393762 .1809791 | .1786943 .4227225 .1786943

q21 | .2414831 .2159794 .0255036 | .8943875 .9457206 .8943875

q22 | .2349718 .0022489 .2327229 **| .0095708** .0978304 .0095708

q17 | .1671258 .0155154 .1516104 | .0928369 .3046915 .0928369

q18 | .1044252 .0050351 .0993901 **| .0482174** .2195847 .0482174

q7 | .2328772 .0027513 .2301259 | **.0118145** .1086945 .0118145

q12 | .2404359 .0147798 .2256561 | .0614708 .247933 .0614708

----------------+--------------------------------------------+------------------------------

overall | | .9864072

------------------------------------------------------------------------------

mc = correlation between depvar and its prediction

mc2 = mc^2 is the Bentler-Raykov squared multiple correlation coefficient

# C. Supplementary data – Attitude Scale EFA

1. Bartlett’s test and KMO

Determinant of the correlation matrix

Det = 0.001

Bartlett test of sphericity

Chi-square = 2554.909

Degrees of freedom = 66

p-value = 0.000

H0: variables are not intercorrelated

Kaiser-Meyer-Olkin Measure of Sampling Adequacy

KMO = 0.840

1. Scree plot


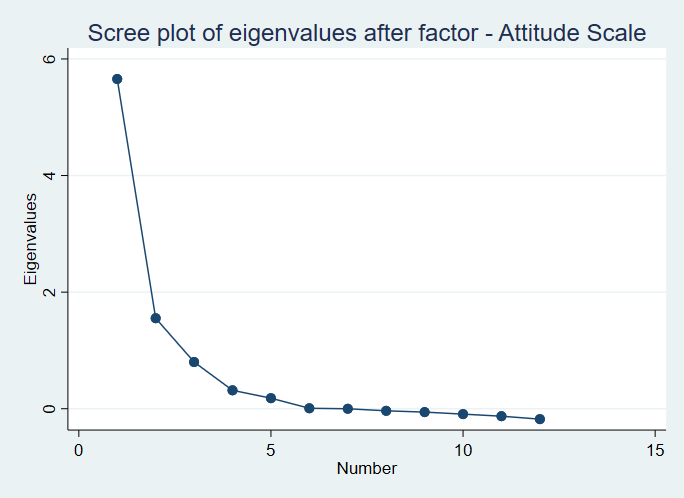


1. Parallel test

Computing: 10% 20% 30% 40% 50% 60% 70% 80% 90% 100%

Results of Horn's Parallel Analysis for principal components

100 iterations, using the mean estimate

--------------------------------------------------

Component Adjusted Unadjusted Estimated

or Factor Eigenvalue Eigenvalue Bias

--------------------------------------------------

1 5.0467878 5.3067225 .25993466

2 1.5861565 1.76043 .17427349

3 1.0680422 1.2177077 .14966547

4 .59085371 .72243513 .13158143

5 .62795089 .67828368 .05033278

6 .58363041 .59363815 .01000774

7 .47959375 .45992481 -.01966894

8 .51082588 .43444795 -.07637793

9 .46852996 .33665206 -.1318779

10 .3529253 .21043766 -.14248765

11 .38787993 .18933638 -.19854355

12 .29682366 .08998398 -.20683968

--------------------------------------------------

Criterion: retain adjusted components > 1

# D. Supplementary data – Attitude Scale CFA

Equation-level goodness of fit

-------------------------------------------------------------------------------------------------------

| Variance |

depvars | fitted predicted residual | R-squared mc mc2

----------------+--------------------------------------------+-----------------------------------------

observed | |

q7 | 1.142332 .4747456 .6675868 | .4155932 .6446652 .4155932

q8 | .9426648 .6185709 .3240939 | .6561939 .810058 .6561939

q9 | .7700901 .2942612 .4758289 | .3821127 .6181526 .3821127

q10 | .8699011 .6363554 .2335458 | .7315261 .855293 .7315261

q11 | .7658099 .4768463 .2889636 | .6226693 .7890939 .6226693

q1 | 1.089634 .9161876 .1734465 | .8408213 .9169631 .8408213

q2 | 1.166769 .9925239 .1742452 | .8506601 .9223123 .8506601

q3 | 1.525048 .3822588 1.142789 | .2506537 .5006533 .2506537

q4 | 1.052858 .4500955 .6027622 | .4274989 .653834 .4274989

q5| .7799255 .4164739 .3634515 | .533992 .7307475 .533992

q6| .7537281 .3178689 .4358592 | .4217289 .6494066 .4217289

q12 | .718773 .2252164 .4935566 | .3133346 .559763 .3133346

-------------+---------------------------------+------------------------------

overall | | .9952808

------------------------------------------------------------------------------

mc = correlation between depvar and its prediction

mc2 = mc^2 is the Bentler-Raykov squared multiple correlation coefficient

---------------------------------------------------------------------------------------
