# Supplementary material for: Exploratory and confirmatory factor analysis of the questionnaire on Palliative Care for Advanced Dementia (qPAD) using a large sample of staff from Australian residential aged care homes
Source: Int J Older People Nurs. 2022 Oct 8;18(1):e12505. doi: 10.1111/opn.12505 (PMC10078386; doi:10.1111/opn.12505)
Supplement: Supplementary file 2 — Table S1 [file OPN-18-0-s003.docx]

Table S1: Exploratory factor analysis with Promax oblique rotation loadings of qPAD Knowledge Test – replicating methods used in original analysis by Long et al. 2012 (N=364)

| **Item no.** | **Knowledge test items** | **Factor 1** | **Factor 2** | **Factor 3** |
| --- | --- | --- | --- | --- |
| 1 | The best way to prevent weight loss for persons with advanced dementia is to keep them on their special medical diets (e.g., low fat, cardiac, renal). | 0.303 | -0.313 | 0.060 |
| 2 | It is possible to prevent pressure ulcers in persons with advanced dementia. | 0.085 | -0.101 | **0.572** |
| 3 | It is possible to prevent weight loss in most persons with advanced dementia. | -0.302 | -0.176 | **0.475** |
| 4 | Since persons with advanced dementia are so impaired, it is not likely that they are depressed. | **0.537** | -0.068 | -0.053 |
| 5 | One benefit of advanced dementia is that people no longer have pain. | **0.618** | 0.023 | -0.141 |
| 6 | When a person is resistive to “hands-on” care, it is best to stop what you are doing and come back later to try to complete the task. | 0.094 | 0.223 | **0.410** |
| 7 | Persons with advanced dementia should take showers just as other persons do. | 0.300 | -0.176 | -0.280 |
| 8 | Persons with advanced dementia cannot verbally tell us when they are hungry or thirsty. | 0.155 | **0.735** | -0.053 |
| 9 | Persons with advanced dementia can reposition themselves easily in their chairs. | **0.619** | 0.261 | -0.162 |
| 10 | Although persons with advanced dementia are incontinent, it is still possible to toilet them. | -0.019 | -0.053 | **0.645** |
| 11 | The sounds of music, meal service and conversations during dining do not generally pose problems for people with advanced dementia. | **0.445** | -0.081 | 0.113 |
| 12 | Persons with advanced dementia typically die from some sort of infection, such as pneumonia or a urinary tract infection. | -0.194 | 0.246 | 0.254 |
| 13 | Physical restraints decrease the chance that a person with advanced dementia will fall. | **0.469** | -0.048 | 0.171 |
| 14 | When people “call out” over and over again, it is best to not worry about this behavior because this is a common occurrence for persons with advanced dementia. | **0.593** | -0.020 | 0.124 |
| 15 | Persons with advanced dementia will never experience boredom. | **0.689** | 0.058 | 0.014 |
| 16 | If persons with advanced dementia resist (e.g., hit, bite, kick etc.) a brief change, it may be due to invasion of privacy. | 0.062 | 0.083 | **0.602** |
| 17 | Persons with advanced dementia should get pain medications around-the-clock, when needed. | 0.175 | **0.441** | 0.288 |
| 18 | “Anticipation of need” refers to addressing the needs of persons with advanced dementia through a daily schedule established by the facility where they live. | -0.087 | -**0.432** | -0.026 |
| 19 | If a person with advanced dementia is unable to sleep at night, a sleeping medication should be considered first. | **0.431** | -0.180 | 0.285 |
| 20 | When persons with advanced dementia spit out their food, it is because they are not hungry. | 0.302 | 0.117 | 0.019 |
| 21 | Persons with advanced dementia really can’t convey or relate to caregivers if they are hungry, have pain, or need to use the bathroom. | 0.113 | **-0.608** | 0.130 |
| 22 | Persons with advanced dementia can fatigue or tire easily, and as a result, they usually need to lie down frequently. | 0.039 | **0.607** | 0.157 |
| 23 | When persons with advanced dementia rapidly become more confused or display changes in behavior, it is likely that their dementia is getting worse. | 0.309 | **-0.451** | 0.149 |

Method: Principal-component factors; Salient loadings (≥0.40) in boldface. 4 items had weak loadings.

**Internal consistency**

Factor 1 (items 4,5,7,9,11,13,14,15,19,20) α=0.72

Factor 2 (items 1,8,17,18,21,22,23) α=0.61

Factor 3(items 2,3,6,10,12,16,) α=0.45
