# Supplementary material for: Exploratory and confirmatory factor analysis of the questionnaire on Palliative Care for Advanced Dementia (qPAD) using a large sample of staff from Australian residential aged care homes
Source: Int J Older People Nurs. 2022 Oct 8;18(1):e12505. doi: 10.1111/opn.12505 (PMC10078386; doi:10.1111/opn.12505)
Supplement: Supplementary file 3 — Table S2 [file OPN-18-0-s001.docx]

**Table S2: Exploratory factor analysis with Varimax orthogonal rotation loadings of qPAD Attitude Scale – replicating methods used in original analysis by Long et al. 2012 (N=364)**

| **Item**  **number** | **Attitude scale statements** | **Factor 1** | **Factor 2** | **Factor 3** |
| --- | --- | --- | --- | --- |
| 1 | I believe my work experience enables me to discuss advanced dementia care with families. | 0.168 | **0.902** | 0.110 |
| 2 | I believe my education enables me to discuss advanced dementia care with families. | 0.177 | **0.917** | 0.068 |
| 3 | I believe it is important that caregivers provide families with information about end-of-life decisions. | 0.214 | **0.642** | 0.207 |
| 4 | Families are given consistent information about the consequences of their end-of-life care decisions. | 0.092 | **0.415** | **0.701** |
| 5 | Families are regularly included in ongoing discussions regarding advanced dementia care needs for their loved ones. | 0.190 | 0.162 | **0.814** |
| 6 | I frequently talk with my teammates about how we can change and improve the care for persons with advanced dementia. | 0.335 | 0.162 | **0.577** |
| 7 | I am regularly included in the care-planning for persons with advanced dementia. | **0.717** | 0.367 | 0.165 |
| 8 | My supervisor and team regularly listen to me regarding suggestions for persons with advanced dementia. | **0.870** | 0.245 | 0.100 |
| 9 | On most days, I am satisfied with my job of caring for persons with advanced dementia. | **0.612** | -0.069 | **0.431** |
| 10 | My input and opinion are valued regarding the needs of persons with advanced dementia. | **0.891** | 0.197 | 0.131 |
| 11 | On most days, I feel I’m part of the care team. | **0.755** | 0.060 | 0.347 |
| 12 | I enjoy providing care for persons who have advanced dementia. | 0.352 | -0.092 | **0.665** |

Method: principal-component factors. Salient loadings ≥0.40 in boldface.

Internal consistency

Factor 1 (items 7-11) α=0.88

Factor 2 (items 1-3) α=0.83

Factor 3 (items 4-6 and 12) α=0.75
